# A non-invasive capacitive sensor to investigate the Leidenfrost phenomenon: a proof of concept study

**DOI:** 10.1038/s41598-024-61222-z

**Published:** 2024-05-08

**Authors:** Abhishek S. Purandare, Jelle Rijs, Pankaj Sagar, Srinivas Vanapalli

**Affiliations:** 1 Applied Thermal Sciences laboratory, Faculty of Science and Technology, University of Twente, Post Bus 217, 7500 AE Enschede, The Netherlands; 2 Department of Instrumentation, Cochin University of Science and Technology, Kochi, Kerala 682022 India

**Keywords:** Physics, Applied physics, Fluid dynamics

## Abstract

A volatile sessile liquid droplet or a sublimating solid manifests levitation on its own vapor when placed on a sufficiently heated surface, illustrating the Leidenfrost phenomenon. In this study, we introduce a non-invasive capacitance method for investigating this phenomenon, offering a potentially simpler alternative to existing optical techniques. The designed sensor features in-plane miniaturized electrodes forming a double-comb structure, also known as an interdigitated capacitor. Initially, the sensor’s capacitance is characterized for various distances between the sensor and a dielectric material. The influence of the sensor substrate material and the spacing between the electrodes on the sensor’s capacitance is also investigated. To demonstrate the feasibility of the method, a sublimating dry ice pellet is placed on the capacitive sensor, and its performance is evaluated. We present results for the dimensionless vapor layer thickness and the pellet’s lifetime at different substrate temperatures, derived from the capacitance output. The results are compared with Optical Coherence Tomography (OCT) data, serving as a benchmark. While the temporal evolution of the sensor’s output, variation in the dimensionless vapor layer thickness, and the lifetime of the dry ice pellet align with expected results from OCT, notable quantitative deviations are observed. These deviations are attributed to practical experimental limitations rather than shortcoming in the sensor’s working principle. Although this necessitates further investigation, the methodology presented in this paper can potentially serve as an alternative for the detection and measurement of Leidenfrost vapor layers.

## Introduction

In the well-known Leidenfrost phenomenon, a volatile liquid droplet hovers over its own vapor when placed on a surface whose temperature is significantly greater than the drop’s boiling temperature. This phenomenon is also observed for solids whose triple point pressure is above ambient pressure, thus preventing the liquid state and causing the solid to continuously sublimate on a surface whose temperature surpasses the sublimation temperature of the solid material. A common example is solid carbon dioxide, popularly known as dry ice, with a triple point pressure and temperature of 5.1 atm and $-56.5\,^\circ \hbox{C}$. At ambient pressure, dry ice sublimates at $-78.5\,^\circ \hbox{C}$ under an environment saturated with $\hbox{CO}_{2}$ vapor. As a result, the rapid vapor generation and overpressure induced by the viscous flow beneath sublimating dry ice can levitate it on a relatively hot surface, similar to the levitation of Leidenfrost drops^1–3^. The presence of a vapor layer beneath an evaporating liquid droplet or a sublimating solid insulates them from the surface on which they are placed, resulting in degradation of the transfer of heat from the surface to the drop and an increase in the lifetime of the drop. Consequently, this phenomenon holds importance in a wide range of thermal engineering applications such as spray cooling, heat treatment of metals, cryopreservation, fuel injection, machining, superconductor cooling, heat exchangers and cleaning^4–10^. While the presence of the vapor layer is favorable in applications involving cleaning, it may lead to the so-called burn-out situation in cooling applications, where cooling efficiency drops significantly, potentially damaging expensive equipment. Therefore, due to its broad implications, quantitative studies involving measurements of the vapor layer thickness beneath Leidenfrost droplets or solids are imperative.

Although critical, measuring vapor layers is challenging as the thickness can be a few micrometers or tens of micrometers, forming very rapidly under a Leidenfrost object^11^. Initial measurements in 1963 employed X-ray imaging to determine vapor layer thickness for a static Leidenfrost water droplet on a heated brass substrate, reporting values of $30\,\upmu \hbox{m}$ and $60\,\upmu \hbox{m}$ at surface temperatures of $200\,^\circ \hbox{C}$ and $500\,^\circ \hbox{C}$, respectively^12^. Chandra and Aziz^13^ measured the temporal evolution of vapor layer thickness beneath a liquid nitrogen droplet on a glass surface using back light imaging, indicating a decrease in thickness over time. Biance et al.^14^ confirmed this evolution using laser diffraction patterns for a Leidenfrost water droplet on a $350\,^\circ \hbox{C}$ duralumin plate. Recent interferometric studies by Burton et al.^15^ and Celestini and Kirstetter^16^ revealed non-uniform vapor layer profiles, contrasting with the assumed uniform thickness in prior studies, as theoretically described by Sobac et al.^17^ using a model lubrication approximation for the static Leidenfrost droplet. In interferometric techniques, the exact vapor layer thickness is unknown without a reference thickness in the system. This challenge is addressed by employing color interferometry with a white light source, as multi-wavelength interference patterns contain information on the absolute thickness of the vapor layers^18,19^. Shriota et al.^20^ recently adapted the frustrated total internal reflection of light technique, developed by Kolinski et al.^21,22^, to investigate layers thinner than the wavelength of the light source, overcoming limitations in studies based on light interference patterns constrained by the wavelength. For Leidenfrost solids, direct measurement of vapor layer thickness was absent until our recent work, which utilized the Optical Coherence Tomography (OCT) technique to elucidate the vapor layer beneath a sublimating dry ice pellet on a temperature-controlled sapphire substrate^23^.

It is evident that the majority of studies have predominantly relied on optical techniques for measuring vapor layers beneath a Leidenfrost object. Beyond their technical limitations, these optical methods require expensive and specialized image acquisition and light source systems, as well as meticulous alignment procedures. Additionally, most of the aforementioned studies have been confined to substrates fabricated from glass or sapphire to establish an optically transparent pathway for light approaching the Leidenfrost object from the bottom of the substrate. Consequently, given these constraints, exploring an electrical technique for investigating Leidenfrost phenomena holds potential due to its simplicity, cost-effectiveness, and recent advancements in electronic devices and fabrication techniques. Taking inspiration from the significance of the capacitance of a system subjected to the electrowetting-on-dielectric phenomenon, where an electrical stimulus across a solid-liquid interface alters the wetting properties of the solid^24–26^, Roques-Carmes et al.^27^ recently proposed a capacitive technique to study the Leidenfrost phenomenon. In this approach, a system comprising a liquid droplet and a hot solid surface is represented by a parallel plate capacitor, with the solid surface and a conductive water droplet forming the capacitor’s plates, and the vapor layer between them serving as the dielectric material. However, for successful experimental outcomes, a stainless steel electrode with a 1 mm diameter was inserted to a depth of 1 mm inside the droplet, preventing its movement on the hot surface. Moreover, capacitance measurements were not feasible throughout the entire droplet’s lifetime, as the measurement became unstable when the droplet’s diameter reduced to twice that of the inserted electrode. Furthermore, the insertion of an electrode into a Leidenfrost object is not universally applicable; for example, in a Leidenfrost solid or in a droplet with a temperature lower than that of the electrode, such as a cryogenic Leidenfrost droplet. Consequently, due to this limitation, we propose a non-invasive capacitive technique based on the fringing field of electrodes aligned with the solid substrate on which the Leidenfrost object is positioned.

In this study, we aim to demonstrate the feasibility of a capacitive sensor featuring a double-comb structure, commonly known as an interdigitated capacitor, for investigating the Leidenfrost phenomenon. Initially, we conduct an analytical analysis of the electrostatic problem, considering a simplified geometry of the capacitive sensor to establish design guidelines for the spacing between the so-called fingers of the comb structure. Prior to assessing the sensor’s performance with an actual Leidenfrost object, a characterization study is undertaken to numerically and experimentally explore the variation in the sensor’s capacitance for various distances between a dummy Leidenfrost object and the sensor. Furthermore, we present the influence of finger spacing and the sensor’s substrate material on the sensor’s output. Finally, we demonstrate the proof of concept by employing a dry ice pellet as the Leidenfrost object, which sublimates on the temperature-controlled capacitive sensor. The study’s results, focusing on the dimensionless vapor layer thickness and the lifetime of the pellet at different substrate temperatures, are then compared to the outcomes obtained from the OCT technique in our previous work^23^.

## Methods

The methodology employed in this study is structured into two parts. In the first part, numerical and experimental approaches are utilized to characterize the capacitance output of the sensor across varying distances between the upper surface of the sensor and the lower surface of a simulated Leidenfrost object fabricated from Teflon. This characterization process aims to establish a correlation between capacitance and the intervening distance. Subsequently, this derived correlation is leveraged in the latter phase of the methodology involving experiments with an actual Leidenfrost object, i.e. dry ice pellet sublimating on the capacitive sensor to accomplish the following objectives: (i) anticipate the temporal evolution of capacitance for a dry ice pellet, (ii) determine the initial values of vapor layer thickness corresponding to different substrate temperatures, and (iii) estimate the lifespan of the authentic Leidenfrost object. These two parts of the methodology are described in the following sections subsequent to the fabrication procedure elucidated for the capacitive sensors employed in this study.

### Sensor fabrication

**Figure 1:**
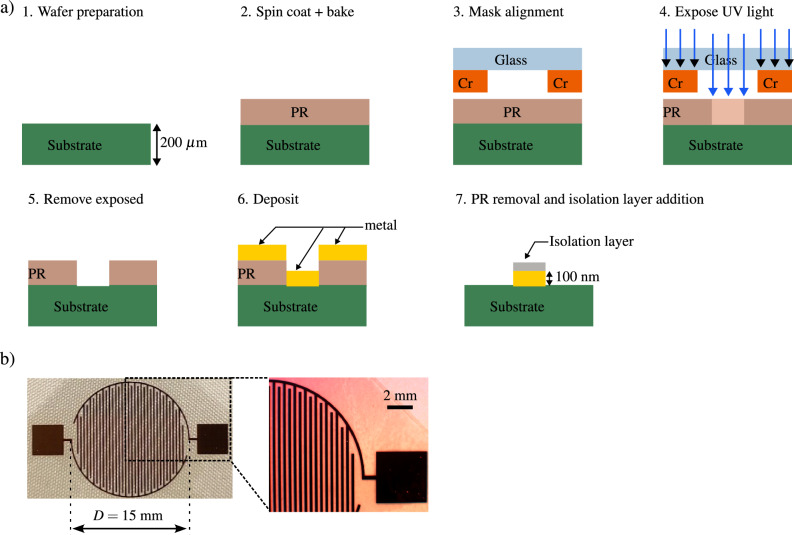
(**a**) Schematic of the steps involved in the fabrication of the capacitive sensor with a glass substrate. (**b**) The sensor with a glass substrate and a finger spacing of $200\,\upmu \hbox{m}$ used to investigate the Leidenfrost phenomenon. The enlarged view shows the comb structure of the sensor.

The capacitive sensors under investigation were produced on two distinct substrate materials: FR4 and glass. External fabrication using the chemical deposition process was employed for the FR4-based sensors. In contrast, in-house development was undertaken for the glass substrate sensors, utilizing the photolithography process illustrated schematically in Fig. 1a. The process initiates with the preparation and cleaning of a glass wafer. Subsequently, a photoresist solution (PR), composed of light-sensitive polymers capable of either forming or breaking bonds upon exposure to UV light (positive or negative resist, respectively), is applied to the wafer using spin coating. The coated photoresist is then subjected to baking to eliminate the solvent, leaving behind the desired photoresist pattern. Once the photoresist is in place, a mask is aligned on the wafer. The mask, typically consisting of a transparent glass layer with a light-blocking material such as chromium (Cr), is employed to define the pattern. For straightforward 2D deposition designs, the pattern is either directly copied onto the mask or created by cutting out the light-blocking material from a full layer, depending on the photoresist type. During exposure, UV light is filtered by the mask, altering the bonds of the photoresist where the light passes through. Subsequent to exposure, the undesired photoresist is removed by immersing the wafer in a developer solution, followed by a cleaning process. The selected metal for the structure is then deposited onto the wafer, with common techniques including evaporation in vacuum, where the metal adheres to the substrate, and sputtering, where ions are accelerated into a target material, releasing atoms towards the wafer. In the last step, the photoresist is removed by chemically dissolving it and finally a layer of isolation made of nitride is added on the metal surface. This isolation layer serves to prevent unwanted chemical reactions at the electrode surface. Additionally, it acts as a physical barrier between the electrodes and the vapor generated by the Leidenfrost object, potentially preventing the vapor from influencing the electrodes. The comprehensive process described in this section ensures the precise fabrication of the comb structure of the sensor on the glass wafer, an example of which is shown in Fig. 1b).

### Setup to characterize sensor output

#### Numerical method

**Figure 2:**
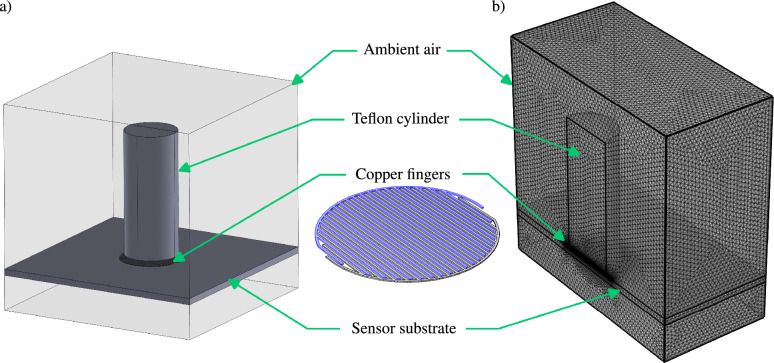
(**a**) Geometry employed in the numerical model to predict capacitance values for several distances between a Teflon cylinder and the sensor. (**b**) Mesh used in simulating half geometry of the numerical model.

To assess the viability of the proposed capacitive method and explore capacitance variation with distance between a dielectric and the sensor, a numerical investigation is conducted using COMSOL Multiphysics 6.0. The 3D geometry employed is illustrated in Fig. 2a, comprising four domains: ambient air ($50 \times 50 \times 50$ mm), FR4 sensor substrate ($50 \times 50 \times 0.2$ mm), Teflon cylinder as the dielectric ($D=12.7$ mm, $H=30$ mm), and two sets of 20 copper fingers with a width and spacing of $\approx 190\,\upmu \hbox{m}$ and a thickness of $20\,\upmu \hbox{m}$, spanning the sensor diameter of 15 mm. The required material properties of all domains are incorporated from the simulation software’s properties library. The electrostatic field in the geometry is solved, applying charge conservation to all domains, with one set of copper fingers set to 1 V and the other to 0 V, forming the boundary conditions for each set of copper fingers. Due to limited computational memory, only half of the geometry is simulated. The four domains numerical domains are discretized using a Tetrahedron mesh having approximately $12\times 10^6$ mesh elements. The mesh resolution is the highest at the copper fingers, gradually increasing towards the outer boundaries of the air domain as shown in Fig. 2b. The capacitance output of the sensor is derived from the charge and electric potential on the copper fingers, and this output is doubled to represent the full geometry. The ratio between the capacitance output for the full and half geometry is approximately two, as validated by simulations for fingers with a thickness exceeding $50\,\upmu \hbox{m}$, allowing simulation of the full geometry without memory constraints. Simulations are conducted to determine the capacitance values for various distances between the top of the copper fingers and the bottom of the Teflon cylinder. Additional simulations explore the influence of the sensor substrate’s thickness and the height of Teflon on the sensor’s output, with detailed results discussed in the results and discussion section.

#### Experimental method

**Figure 3:**
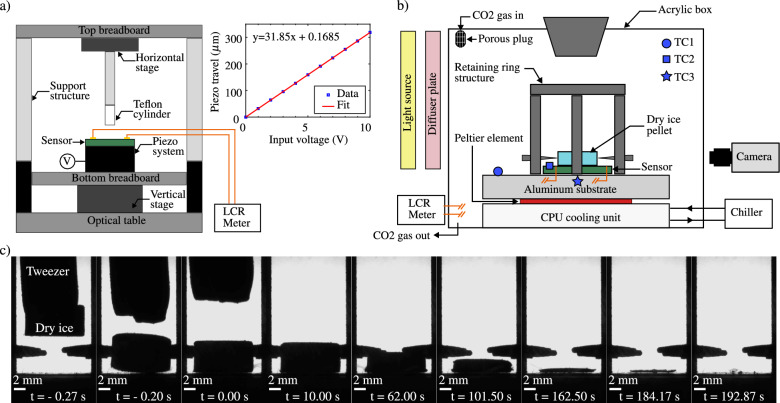
(**a**) Schematic of the experimental setup used for characterizing sensor capacitance as a function of the distance between the sensor and the Teflon cylinder. (**b**) Schematic of the experimental setup used to test the feasibility of the capacitive sensor for investigating the Leidenfrost phenomenon of a dry ice pellet. (**c**) An example image sequence of the dry ice pellet sublimating on the capacitive sensor during an experimental run.

The schematic of the experimental setup for characterizing the capacitance output of the sensor in relation to the distance from a Teflon dielectric material is depicted in Fig. 3a. The actual image of the experimental setup can be found in supplementary Fig. 1a, shown in the supplementary material of this work. A Piezosystemjena PZ400 piezoelectric positioning stage, offering a range and resolution of approximately $320\,\upmu \hbox{m}$ and 0.8 nm, controls the vertical movement of the sensor, mounted atop the piezo system. The Teflon cylinder is affixed to a horizontal displacement stage on the top breadboard. Voltage and current supplied to the piezo system, managed by a d-Drive controller with an amplifier, are regulated, and the corresponding vertical movement is recorded using a LABVIEW program. The relationship between applied voltage and vertical displacement of the piezo system, calibrated with a Millimar C1208 length measuring instrument, is illustrated in Fig. 3a. For initial positioning, a bottom vertical translation stage (Melles Griot 07 TEZ004) is employed, and a top horizontal translation stage (ThorLabs MT1A/M) centers the Teflon cylinder relative to the sensor before the commencement of the characterization experiment. The capacitance output of the sensor is measured utilizing a precision LCR meter (LCR-6200 by GW Instek) integrated with a data acquisition system developed in LABVIEW with an accuracy of approximately 0.2% for capacitance values within the range investigated in this study. The measurement procedure involves supplying an AC signal with a fixed frequency of 20 kHz into the device under test, after which the resulting current passing through the device is detected by the meter. Utilizing amplitude, phase, and frequency data obtained from the resulting current and excitation voltage, the meter computes the capacitance. To mitigate the influence of spurious noise signals, the measurement setup incorporates suitable shielding measures.

It is important to note that during capacitance measurements with a Leidenfrost object, there is a possibility of movement of the object in the plane of the sensor. Despite selecting a sensor diameter larger than the Leidenfrost object and employing a retaining structure to prevent sensor movement and maintain its approximate centering, concerns regarding the edge effects of the sensor on its output may arise. To investigate this effect, the horizontal displacement of the Teflon cylinder is varied, and capacitance is recorded while maintaining a constant distance of $50\,\upmu \hbox{m}$ between them. Only a minor change in capacitance, 1% of its base value, is measured when the Teflon cylinder is displaced from the center of the capacitive sensor towards its edge. This indicates that the edge effect of the sensor has a negligible influence on the absolute values of capacitance changes measured by the sensor.

### Setup to investigate the Leidenfrost phenomenon

The feasibility of utilizing the capacitive sensors for investigating the Leidenfrost phenomenon is demonstrated through an experimental setup, as schematically shown in Fig. 3b. The actual image of the experimental setup can be found in supplementary Fig. 1b, shown in the supplementary material of this work. The capacitive sensor with 200 $\upmu$m glass substrate, is affixed to a 1 cm thick aluminum substrate using thermal paste. Temperature control of the bottom face of the aluminum substrate is achieved by modulating the power supply to a Peltier element thermally bonded to it. Another side of the Peltier element is connected to a temperature-controlled CPU cooling unit (Alphacool Eisblock XPX), sustained by a cooling fluid based on silicon oil. The cooling fluid temperature is regulated by an external liquid chiller (IKA HRC 2 control). A 3D printed retaining ring with eight legs, each equipped with a Nylon pin, constrains the movement of the dry ice pellet during its sublimation on the capacitive sensor.

To produce dry ice pellets, first dry ice in the form of snow with a powder-like structure is generated by throttling high-pressure liquid $\hbox{CO}_{2}$ (approximately 57 bar at around $20\,^\circ \hbox{C}$) through an orifice using a commercially available apparatus known as CARBONEIGE 100, manufactured by Air Liquide. Subsequently, the dry ice snow is die-forged into small pellets with a diameter (*D*) of 10 mm and a height (*H*) of 5 mm. The effective density of the pellets, determined during experiments from their initial mass and volume, falls within the range of approximately 760–1015 $\hbox{kg}/\hbox{m}^{3}$. To prevent moisture condensation on the dry ice pellets from the ambient air, the setup is enclosed in an acrylic box, continuously flushed with $\hbox{CO}_{2}$ gas through a porous plug to mitigate the impact of flow on pellet sublimation. The flushing also saturates the pellet’s surroundings with $\hbox{CO}_{2}$ gas, minimizing sublimative cooling due to concentration gradient at the sublimating interface of the pellet^28^. The $\hbox{CO}_{2}$ gas is expected not to affect the capacitance output of the sensor, given its similarity in permittivity to air. It is noteworthy that although precautions are taken to minimize condensation and freezing of ambient moisture on the dry ice pellets during experiments by purging the test section with $\hbox{CO}_{2}$ gas, there remains a possibility that moisture from the air may condense and freeze on small particles of dry ice snow produced during the expansion of liquid $\hbox{CO}_{2}$. Consequently, water-ice particles could be trapped inside the dry ice pellets, and during the sublimation of the pellets, these water-ice particles may intermittently reach the sensor’s surface, leading to local peaks in the sensor’s capacitance values. Despite this limitation, the overall temporal evolution of the sensor’s capacitance during the sublimation of a dry ice pellet on top of it remains unaffected and measurable. As shown later in the subsequent sections, the result from the capacitance method proposed in this work is compared to that of an optical coherence tomography technique, which is based on a Michelson interferometer where the two light beams, namely the reference and sample beam, interfere and gives the measure of the changes in the path length of the light beams, allowing for precise measurements of the vapor layer thickness below a Leidenfrost object. Details of the experimental setup and procedure employed to measure the vapor layer thickness below a disc-shaped dry ice pellet can be found in^23^.

Before commencing an experiment, the cooling unit, aluminum substrate, and consequently, the sensor substrate are brought to the desired temperature using appropriate PID settings in the LABVIEW program. A dry ice pellet is then carefully placed on the capacitive sensor from the top of the acrylic cover using insulated-tipped tweezers. Simultaneously, the LABVIEW program records capacitance output and temperatures at various locations on both the aluminum and sensor substrates. The SpinView software captures images of the dry ice pellet. All three datasets are collected continuously until the completion of the pellet’s sublimation. Type E thermocouples measure temperatures at the top face of aluminum (TC1) and sensor (TC2) substrates, as well as at a location 0.5 mm below the top of the aluminum substrate (TC3). The steady-state temperatures at these locations before the experiment showed minimal deviations, reducing by a maximum of $\approx$ 0.5 K during the dry ice pellet sublimation. The sensor’s capacitance output is measured using an LCR meter with settings similar to those in the characterization study mentioned in the previous section. Images of the dry ice pellet during the experiment are captured using a FLIR (ORX-10GS-123S6) camera. An illustrative sequence of images depicting the sublimation of the dry ice pellet on the sensor at a temperature $T_p = 303$ K is presented in Fig. 3c, with time stamped using MATLAB R2022b.

## Results and discussion

In this section, a design guideline for the spacing between two electrodes of the comb capacitive sensor is initially established. Subsequently, numerical and experimental results are provided regarding the characterization of the sensor’s capacitance for a dummy Leidenfrost object, specifically a Teflon cylinder. Finally, the results illustrating the validation of the proposed capacitance method are presented for the scenario of a disc-shaped dry ice pellet sublimating on the temperature-controlled capacitive sensor.

### Sensor design

**Figure 4:**
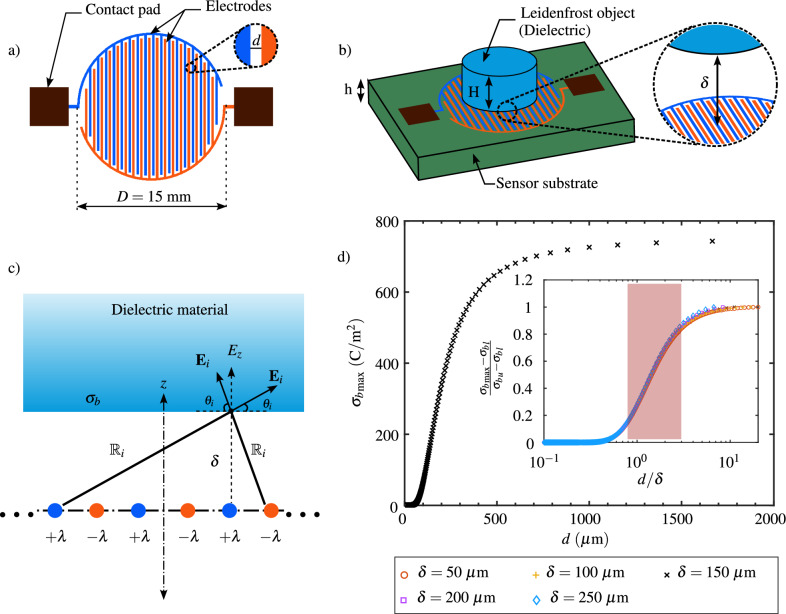
(**a**) 2D schematic of the capacitive sensor with double comb structure (**b**) Schematic representation of the situation where a Leidenfrost object is placed on the capacitive sensor with a Leidenfrost layer in between the two. (**c**) Schematic of the simplified geometry considered for the analytical description of a dielectric above infinite line charges. (**d**) Maximum value of the surface charge density on the bottom surface of the dielectric as a function of the spacing between two consecutive fingers of the comb structure of sensor. The snippet of the figure represents the dimensionless surface charge density as a function of the dimensionless spacing between the fingers of the sensor.

The 2D configuration of the capacitive sensor utilized in this study is depicted in Fig. 4a. The sensor comprises two in-plane electrodes, each featuring fingers alternating to form a double comb-like structure. The spacing between consecutive fingers is denoted as *d*, and the overall sensor diameter is *D*. Contact pads on each electrode facilitate the connection between the sensor and an LCR meter to measure the sensor’s capacitance output. A thin substrate with a thickness of *h* supports the sensor electrodes. On top of this substrate, a Leidenfrost object with a height *H* can be placed, and a vapor layer with a thickness $\delta$ separates the object from the sensor, as schematically shown in Fig. 4b.

This problem is first analytically analyzed to gain insights into the physical functioning of the sensor and establish design guidelines for the finger spacing (*d*) necessary for its application in investigating the Leidenfrost phenomenon. In this analytical approach (Fig. 4c), the fingers of each electrode are assumed as infinite lines with a line charge density of $+\lambda$ and $-\lambda$ on the positive and negative electrode, respectively. When a homogeneous isotropic Leidenfrost object, acting as a solid dielectric material, is at a distance $\delta$ above the line charges, the electric field ${{\textbf {E}}}$ of the line charges induces electric dipole moments in the dielectric material. In the bulk of the dielectric, electric neutrality is conserved in the case of uniform polarization. However, a thin layer at the bottom surface of the dielectric acquires non-zero net densities $\sigma _b$ of bound charges, which is proportional to the dipole moment per unit volume, **P**, perpendicular to the surface of the polarized dielectric^29^:

1 $$\begin{aligned} \sigma _b = {{\textbf {P}}}\cdot {\hat{{{\textbf{n}}}}}. \end{aligned}$$

The polarization in a homogeneous linear dielectric material in a not too strong electric field, **E**, is given as^29^:

2 $$\begin{aligned} {{\textbf {P}}} = \varepsilon _0\chi {{\textbf {E}}}. \end{aligned}$$

Where $\varepsilon _0$ and $\chi$ are the permittivity of free space and the electric susceptibility of the dielectric material, respectively. In reference to Fig. 4c, the dipole moment in the *z* direction is calculated from the total electric field normal to the dielectric surface ($E_z$), which is basically the electric field due to bound charges and all line charges. While the former’s contribution is $-\sigma _b/2\varepsilon _0$^29^, the $z-$component of the electric field of line charge density is evaluated by superimposing all contributions at different locations on the dielectric surface, as shown in the following equation:

3 $$\begin{aligned} P_z = \varepsilon _0\chi \left( \sum _{i = 1}^{n}\frac{1}{2\pi \varepsilon _0}\frac{\pm \lambda }{{\mathbb {R}}_i}\sin \theta _i - \frac{\sigma _b}{2\varepsilon _0}\right) . \end{aligned}$$

Where *n* is the number of line charges, ${\mathbb {R}}$ is the distance between a line charge and a point on dielectric surface, and $\theta$ is the angle made by a electric field vector with the horizontal. By combining Eqs. (1) and (3), the expression for the bound charge density is obtained:

4 $$\begin{aligned} \sigma _b = \frac{\chi }{\pi (2+\chi )}\left( \sum _{i = 1}^{n}\frac{\pm \lambda }{{\mathbb {R}}_i}\sin \theta _i\right) . \end{aligned}$$

Using the above equation, the maximum value of the bound surface charge density (${\sigma _b}_{\textrm{max}}$) over the bottom surface of the dielectric, at a distance assumed at $150\,\upmu \hbox{m}$ above the line charges, evaluated for different *d* values ranging from $15\,\upmu \hbox{m}$ to 1.6 mm is shown in Fig. 4d. Here, the parameters ${\mathbb {R}}$ and $\theta$ are geometrically evaluated, the magnitude of the line charge density is considered to be $\pm 1~\hbox{C}/\hbox{m}$, and the susceptibility of the dielectric, $\chi =\varepsilon _r-1$, is evaluated to be 1.1 by assuming the relative permittivity of $\varepsilon _r \sim 2.1$. This value of relative permittivity is used as it is expected to represent the permittivity of the Leidenfrost object, i.e., dry ice, which is utilized in the later section to demonstrate the sensor’s feasibility^30^.

It can be observed from Fig. 4d that for a given value of $\delta$, the value of ${\sigma _b}_{\textrm{max}}$ increases with increasing value of *d*, except for very small and large values, where it saturates. A similar trend is observed for different values of $\delta$ as shown in the snippet of the figure representing the variation of bound charge density, normalized by the difference between its upper (${\sigma _b}_u$) and lower (${\sigma _b}_l$) saturated values, over the spacing between the line charges, normalized by the distance between the dielectric and the line charges. It can be seen that all the curves approximately overlap, and the normalized bound charge density starts to saturate for $d < 0.8\delta$ and $d > 3\delta$. Therefore, to have reasonable sensitivity of the sensor, it is recommended that the value of *d* lies in between these bounds, i.e., the value of *d* needs to satisfy the condition $0.8\delta<d<3\delta$ as shown by the shaded region in the snippet of Fig. 4d. Alternatively, for a sensor with given value of *d*, the values of $\delta$ for which it would have reasonable sensitivity is $0.3d<\delta <1.25d$. Hence, to be able to detect the $\delta$ values of a few tens of micrometers to a few hundreds of micrometers below a Leidenfrost object, a sensor with $d=200\,\upmu \hbox{m}$ is fabricated. But, at first, to comprehend the nature of the sensor’s output and how it is influenced by the sensor substrate material and finger spacing, further analysis is carried out in the following section.

### Sensor characterization

The analytical model presented in the preceding section provided a design guideline for the spacing between the fingers of the sensor, assuming negligible finger width and infinite length with a uniform line charge distribution. However, to estimate the magnitude of the change in capacitance expected when a Leidenfrost object is in the vicinity of the sensor, a numerical and experimental study is conducted, taking into account the actual geometry of the sensor. The sensor’s output i.e. its capacitance is characterized for various precisely controlled distances between the sensor and a dielectric material, specifically a Teflon cylinder acting as a dummy Leidenfrost object. Teflon is chosen as the dielectric because its relative permittivity, $\varepsilon _r\sim 2$, is close to that of a dry ice pellet produced by compacting dry ice snow^30,31^, which is later used in the proof-of-concept experiments. Therefore, it is expected that at least the order of magnitude of the change in capacitance as a function of distance will be similar for Teflon and dry ice disks.

#### Variation of capacitance with distance

**Figure 5:**
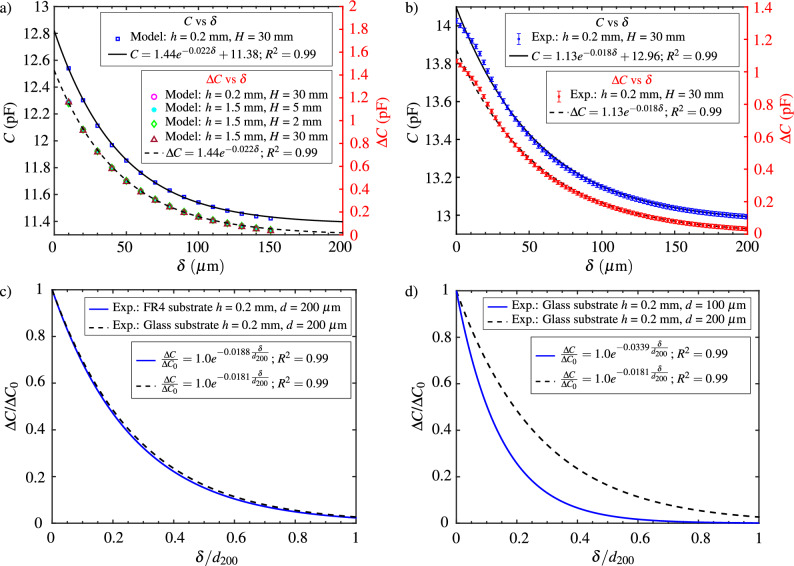
(**a**) Capacitance (left y-axis) and change in capacitance in reference to base capacitance (right y-axis) as a function of the distance between a Teflon cylinder (dielectric) and the sensor, predicted for different values of dielectric height and sensor substrate height. Solid and dashed lines represent the fitted curves. (**b**) Measured values of capacitance (left y-axis) and change in capacitance in reference to base capacitance (right y-axis) as a function of the distance between a Teflon cylinder and the sensor. Solid and dashed lines represent the fitted curves. (**c**) Influence of the sensor substrate material on the variation of the measured values of the dimensionless change in capacitance as a function of the dimensionless distance between the sensor and the dielectric. (**d**) Influence of finger spacing on the variation of the measured values of the dimensionless change in capacitance as a function of the dimensionless distance between the sensor and the dielectric.

The geometry of the investigated sensor in this section comprises an overall diameter of 15 mm, a thickness (*h*) of $200\,\upmu \hbox{m}$, 20 copper fingers per comb, and finger width and spacing of approximately $200\,\upmu \hbox{m}$. The sensor’s capacitance output is examined as a function of the distance ($\delta$) between it and a Teflon cylinder (height $H = 30$ mm, diameter approximately 12 mm). The values of capacitance (*C*) and the capacitance with reference to its base value ($\Delta C$) are predicted using the commercial finite element simulation software COMSOL Multiphysics for various values of $\delta$, as shown in Fig. 5a. The predicted value of *C* is highest at the lowest $\delta$, exponentially decreasing as $\delta$ increases. The exponential decline in capacitance can be attributed to the variation in electric field strength, which is highest between the copper fingers and decreases exponentially as the distance from the copper fingers increases. As the Teflon cylinder traverses within this exponentially decaying field, the capacitance between the two diminishes proportionally with the increasing distance. The predicted nature of sensor capacitance decrease is described by the fitting equation, $C=Ae^{-B\delta }+C_b$ (values shown in Fig. 5a). Here, $C_b$ is the capacitance value when $\delta \rightarrow \infty$ (indicating the sensor’s base capacitance). *A* represents the maximum change in capacitance from the base value ($\Delta C = C-C_b$) when $\delta \rightarrow 0$, and *B* scales with the dielectric’s permittivity and inversely with a length scale attributed to the spacing between copper fingers. Since the change in sensor output when the Leidenfrost object is placed on it is significant, predicted values of $\Delta C$ are also evaluated and shown in Fig. 5a (right y-axis). A total change of $\sim 1.4\,\hbox{pF}$ is predicted when the Teflon cylinder approaches the sensor’s surface. Furthermore, the outcomes from additional simulations conducted with varying combinations of sensor substrate thickness and Teflon cylinder height exhibit comparable variations of $\Delta C$ with $\delta$, as illustrated by the overlap of symbols in Fig. 5a. This suggests that the influence of the geometric thickness of the sensor substrate and the dielectric height on the sensor’s output can be neglected.

The numerical model results revealed an expected order of magnitude for capacitance ranging from a few picofarads to tens of picofarads when a dielectric material is in proximity to the capacitive sensor. The sensor’s capacitance values were measured using an LCR meter (GW Instek LCR-6200), capable of accurate measurements within this range, and are presented in Fig. 5b. The experimental setup details for characterizing the sensor’s output at various distances from the Teflon cylinder are outlined in the Methods section. Data points in Fig. 5b represent the average of three different measurement runs, and error bars indicate the ± standard deviation at a given $\delta$ value. The observed variation of the measured capacitance (*C*) and change in capacitance with respect to the base value ($\Delta C$) with $\delta$ closely resemble the predicted trends in Fig. 4a. Specifically, their variations can be effectively described by the fitting curves $C=Ae^{-B\delta }+C_b$ and $\Delta C=Ae^{-B\delta }$. However, the fitting parameters *A*, *B*, and $C_b$ for the measured values deviate from those of the predicted values as seen from Fig. 5a and b. The value of the parameter $C_b$ is larger in the experimental data as the base capacitance due to the contact pads, solder junctions, and connecting wires is not considered in the simulations. The observed higher value of the parameter *A* in the predicted results compared to the measured values may be attributed to potential misalignment between the Teflon cylinder and the sensor within the experimental setup. Specifically, as the Teflon cylinder approaches the sensor’s surface ($\delta \rightarrow 0$), a minor inclination between the two result in a collision between a part of the edge of the Teflon’s bottom surface and the sensor, impeding the sensor’s movement. This collision induces a reduction in the slope of capacitance near zero values of $\delta$, as illustrated in Fig. 5b. Consequently, the parameter *A* (equivalent to $\Delta C$ as $\delta \rightarrow 0$) reduces in the experimental results. The discrepancy in the value of the fitting parameter *B* between the model and the experiments is ascribed to the variation in spacing between the fingers assumed in the model and the actual manufacturing conditions. The manufactured sensors have a larger spacing between fingers compared to the assumed model, differing by approximately $\sim 10\,\upmu \hbox{m}$. As mentioned earlier, since the parameter *B* inversely varies with finger spacing, the value of this parameter in the experiments is smaller than that in the predicted results. Consequently, this leads to a less steep fitting curve for the measured capacitance values compared to the curve fitting the model data, as the slope of the fitting curve, $dC/d\delta = -ABe^{-B\delta }$, is proportional to the parameter *B* for a given *A* and $\delta$. Despite these observed deviations, the results from the characterization study in this section offer valuable insights into the order of magnitude and the nature of the capacitance variation one can anticipate when a Leidenfrost object, having permittivity similar to that of a Teflon cylinder, levitates over the surface of the designed capacitive sensor.

#### Influence of substrate material

In the preceding section, an analysis was conducted to characterize the output of the capacitive sensor, specifically the capacitance (*C*) and the change in capacitance with respect to the base value ($\Delta C$), in relation to the separation distance ($\delta$). This investigation focused on a sensor equipped with copper fingers as its sensing elements, fabricated on a printed circuit board (PCB) made from a composite material known as FR4, which forms the sensor’s substrate. To assess the influence of the substrate material, experiments were also conducted on a sensor with copper fingers fabricated on a glass wafer. All other parameters, such as the dimensions of the substrate, Teflon cylinder, copper fingers, and spacing between them, remained consistent with those of the sensor with an FR4 substrate.

The variation of $\Delta C$ with $\delta$ for the fitted experimental data obtained from sensors with FR4 and glass substrates is presented in Fig. 5c. The results are expressed in dimensionless form, where $\Delta C$ is normalized with its value at $\delta = 0$ ($\Delta C_0$) for the respective substrate material, and $\delta$ is normalized by the finger spacing, $d_{200} = 200\,\upmu \hbox{m}$. Notably, the results in Fig. 5c exhibit substantial overlap for both sensor substrate materials. This overlapping trend suggests that the substrate material does not significantly influence the nature of the capacitance variation with $\delta$, provided all other parameters related to sensor geometry, dielectric material, and geometry remain constant. Although the magnitude of capacitance, *C*, may change with alterations in substrate material, its value concerning the base value remains approximately independent of the substrate material. Since this study is primarily concerned with the change in capacitance concerning the base value when a Leidenfrost object is placed on the sensor, the influence of the substrate material on electrical quantities, such as capacitance, can be neglected. However, from a heat transfer perspective, it is advantageous for the sensor substrate to exhibit relatively good thermal properties and establish effective thermal contact between the sensor and the surface on which it is placed to minimize temperature gradients during an experiment. The substrate material cannot be metallic or semiconductor due to the risk of short-circuiting the copper fingers of the sensor. Although short-circuiting can be avoided by incorporating a thin insulating film between the fingers and the substrate material, selecting a metal or semiconductor as the substrate would significantly increase the base capacitance. For instance, it may increase by three orders of magnitude, falling in the range of 1–10 s nF, as the metal substrate forms a capacitor with sensor electrodes. This increase poses a challenge in detecting a change in capacitance of 0.1–10 s pF when a Leidenfrost object is placed on the sensor. Sapphire, an electrical insulator with reasonable thermal properties, could serve as a viable alternative to conductors. However, due to the available fabrication techniques, the sensors with FR4 and glass substrates were the focus of analysis in this work. Given that glass exhibits reasonably higher thermal diffusivity ($\alpha _{\textrm{glass}}\approx 0.8\,\hbox{mm}^{2}/\hbox{s}$ at 298 K^32^) compared to FR4 material ($\alpha _{\textrm{FR4}}\approx 0.3\,\hbox{mm}^2/\hbox{s}$ calculated from its thermal conductivity (0.23 W/m K) density ($1900\,\hbox{kg}/\hbox{m}^{3}$) and specific heat capacity ($393\,\hbox{J}/\hbox{kg K}$)^33^), the final experiments involving the Leidenfrost object were conducted using the sensor with a glass substrate.

#### Influence of finger spacing

To evaluate the influence of finger spacing, two sensors with glass substrates featuring different finger spacings, namely $d_{200}=200\,\upmu \hbox{m}$ and $d_{100}=100\,\upmu \hbox{m}$ are experimentally investigated. All other geometric parameters related to the sensors and the Teflon cylinder are similar for the two sensors. The output of both sensors, fitted to the measured values of $\Delta C$ for various values of $\delta$, is presented in Fig. 5d in dimensionless form. Similar to the results of the previous subsection, $\Delta C$ and $\delta$ are made non-dimensional using $\Delta C_0$ and $d_{200}$, respectively. Figure 5d illustrates that, with increasing $\delta$ values, the output of the sensor with $100\,\upmu \hbox{m}$ spacing experiences a rapid decrease, in contrast to the sensor with $200\,\upmu \hbox{m}$ spacing, where a gradual decrease in capacitance output is observed. In other words, for a lower value of *d*, the magnitude of the slope of the curve representing the sensor’s output variation with $\delta$ is higher. As stated in previous sections, the slope of the curve is governed by the parameter *B* in the fitting equation. This implies that for lower *d* values, the fitting parameter *B* is higher, and it scales inversely with the finger spacing of the sensor. Furthermore, it is observed that while the output of the sensor with $100\,\upmu \hbox{m}$ finger spacing approaches zero at $\delta \approx 0.7*d_{200}$ (or at $\delta \approx 1.4*d_{100}$), this occurs at $\delta \approx 1.3*d_{200}$ for the sensor with $200\,\upmu \hbox{m}$ finger spacing. This indicates that values of $\delta$ at or higher than these for respective sensors cannot be accurately measured, aligning with the upper design limit ($\delta <1.25d$) obtained from the results of the analytical model presented in previous section. Therefore, choosing the appropriate finger spacing for the sensor is crucial for the specific application under consideration. For the subsequent experiments with an actual Leidenfrost object discussed in the following section, the sensor with with $200\,\upmu \hbox{m}$ finger spacing is employed, as its output is anticipated to be sensitive to the expected orders of the vapor layer thickness beneath the Leidenfrost object.

### Proof of concept

After comprehending the physical principle of the capacitive measurement technique, assessing the order of magnitude of $\Delta C$ for a dummy Leidenfrost object (i.e., Teflon cylinder), and examining its variation for several values of $\delta$ in the previous section, this section aims to demonstrate the feasibility of the capacitive sensors presented in this work for investigating the Leidenfrost phenomenon of a sublimating dry ice disc.

#### Evolution of capacitance for a dry ice pellet

**Figure 6:**
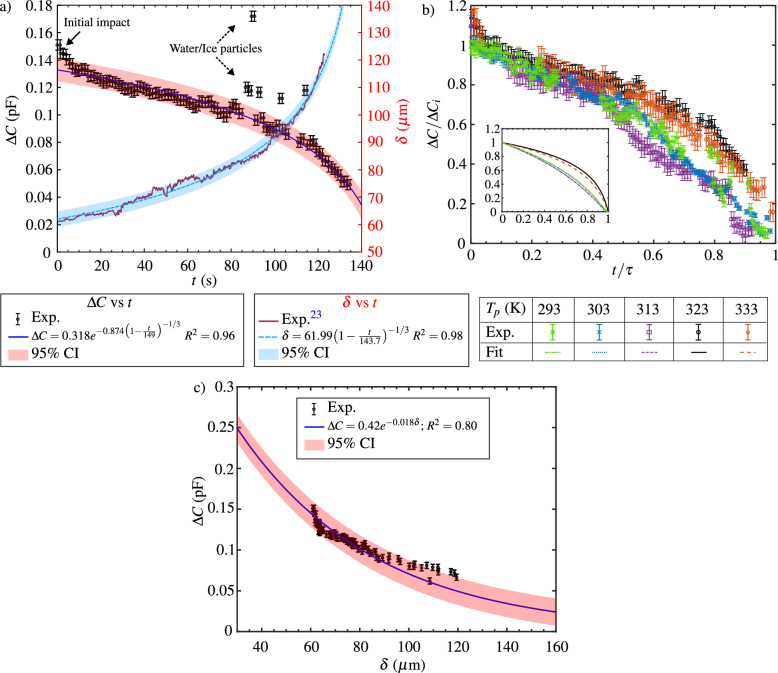
(**a**) Measured temporal evolution of capacitance change of the sensor when a disc-shaped dry ice pellet is sublimating on the sensor. (**b**) Measured variation of dimensionless capacitance change with dimensionless time for the dry ice pellet sublimating on the sensor for different substrate temperatures. (**c**) Measured values of change in capacitance in reference to base capacitance as a function of the vapor layer thickness below a dry ice pellet. This result is derived from (**a**) by considering values of capacitance and the vapor layer thickness at similar time steps.

A disc-shaped dry ice pellet with an initial diameter $D_{i}=10\,\hbox{mm}$, height $H_{i}=5\,\hbox{mm}$ and mass $m\approx 390\,\hbox{mg}$ is placed on the capacitive sensor that is thermally glued to the top surface of a temperature controlled hot aluminium substrate. Consequently, due to the continuous sublimation, the dry ice pellet hovers on a cushioning layer of its own vapor until it completely sublimates and reaches the end of its lifetime ($\tau$). During the pellet’s lifetime, the output of the capacitive sensor is continuously measured using an LCR meter as described in the Methods section. The temporal evolution of the $\Delta C$ measured during the sublimation of the dry ice pellet when the aluminum substrate is maintained at $T_p\approx 323\,\hbox{K}$, is shown in Fig. 6a. The result of the temporal evolution of the vapor layer thickness measured using the Optical Coherence Tomography (OCT) technique for a dry ice pellet ($\approx 395\,\hbox{mg}$) sublimating on a sapphire substrate maintained at $T_p\approx 323\,\hbox{K}$ is also shown in Fig. 6a. As expected, the capacitance reduces over time as the vapor layer thickness below a disc-shaped dry ice pellet increases as the pellet sublimates. Furthermore, it is shown in our previous work^23^ that the vapor layer thickness, for the majority lifetime of the pellet, can be analytically described by:

5 $$\begin{aligned} \delta (t)=\delta _0\left( 1-\frac{t}{\tau }\right) ^{-\frac{1}{3}}, \end{aligned}$$

where $\delta _0$ and $\tau$ are the initial vapor layer thickness at $t=0$, and the characteristic time constant representing the lifetime of the pellet, respectively. They are described as:

6 $$\begin{aligned} \delta _0=\left( \frac{3}{2}\frac{\bar{\mu _v}\bar{k_v}\Delta T}{L\rho _sgH_{i}\rho _{v,sat}}\right) ^{\frac{1}{4}}\left( \frac{D_i}{2}\right) ^{\frac{1}{2}} \end{aligned}$$

and

7 $$\begin{aligned} \tau = \frac{4}{3}\left( \frac{3}{8}\frac{\bar{\mu _v}}{g\rho _{v,sat}}\right) ^{\frac{1}{4}} \left( \frac{D_i^{\frac{2}{3}}\rho _sH_{i}L}{\bar{k_v}\Delta T}\right) ^{\frac{3}{4}}. \end{aligned}$$

The parameters that govern Eqs. (6) and (7) are related to the geometry of the pellet ($D_i$, $H_i$), thermodynamic parameters (*L*: Latent heat of sublimation of dry ice; $\Delta T$: Temperature difference between the sensor and the dry ice pellet; $\rho _s$: Pellet density; $\rho _{v,sat}$: $\hbox{CO}_{2}$ vapor density at sublimation temperature), fluid dynamic properties ($\bar{\mu _v}$: Vapor viscosity; $\bar{k_v}$: vapor thermal conductivity both evaluated at the mean temperature of dry ice and the sensor), and *g* which is the acceleration due to gravity. Note that the Eq. (5) through Eq. (7) have been directly excerpted from our previously published work. For a detailed derivation of these equations, it is recommended to refer to Section “Results and discussion”, “Formulation of the Model,” in the paper by Purandare et al.^23^.

A curve, described by Eq. (5), fitting the measured temporal evolution of the vapor layer thickness, along with the 95% confidence interval, is also shown in Fig. 6a. Equation (5) is substituted in the fitting equation, $\Delta C = Ae^{-B\delta }$, obtained from the characterization study discussed in the previous section. After substitution and grouping the constants (*A*, *B*, $\delta _0$, and $\tau$) a theoretical relationship for the temporal evolution for the change in capacitance is obtained as:

8 $$\begin{aligned} \Delta C = A'e^{-B'\left( 1-\frac{t}{C'}\right) ^{-1/3}}. \end{aligned}$$

Where $A'$, $B' = B\delta _0$ and $C'$ are the fitting parameters for the curve representing the temporal evolution of the change in capacitance when a dry ice pellet is sublimating on top of the capacitive sensor. The curve, represented by the Eq. (8), fitting the measured data of the temporal evolution of change in capacitance along with the fitting parameters and their 95% confidence interval (CI) is shown in Fig. 6a. Since Eq. (8) fits reasonably with the measured data of capacitance, it confirms that the nature of temporal evolution of the change in capacitance can be indicative of the temporal evolution of the vapor layer thickness between the dry ice pellet and the sensor. The peak in the values of $\Delta C$ measured at the initial time indicated in Fig. 6a by a solid arrow is attributed to the initial impact of the dry ice pellet on the sensor. Specifically, at the start of an experiment, the impact of the pellet during its placement on the sensor might squeeze the vapor layer and reduce its thickness between them before the pellet stabilizes on top of the sensor. This reduction in the vapor layer thickness for a small amount of time at the start of an experiment consequently may lead to relatively higher values of $\Delta C$. The outliers shown by the dashed arrow on Fig. 6a are ascribed to a momentary increase in capacitance due to impurities like small water droplets or water-ice particles on the sensor’s surface. These droplets or ice particles can originate from the atmosphere upon condensation and freezing on the dry ice surface or they might be present inside the dry ice pellet during its manufacture, which in both cases eventually accumulate on the sensor’s surface as the dry ice sublimates over time. Consequently, due to the permittivity of water and ice being relatively higher than that of the dry ice, the values of capacitance measured by the sensor intermittently peak during the lifetime of the dry ice pellet. This undesirable effect sometimes can be more prevalent towards the end of the lifetime of the pellet potentially leading to large peaks in capacitance and missing data points towards the end of experiments as evident in Fig. 6a. The error bars shown for the measured values are of the order of 0.004 pF which is evaluated by considering the biggest of the values corresponding accuracy factor of the LCR meter and the offset in the base value of $\Delta C$ due to moisture deposition at the end of an experiment when the dry ice pellet has sublimated.

Despite the practical limitations encountered in the experimental setup, an examination of the temporal evolution of capacitance change reveals a consistent pattern across five distinct substrate temperatures, as illustrated in Fig. 6b. Here, $\Delta C$ has been non-dimensionalized with respect to its initial value ($\Delta C_i$ at $t = 0$ s), and time has been non-dimensionalized by the pellet’s lifetime. Ideally, a complete overlap of all curves is anticipated. However, upon closer examination of the snippet from Fig. 6b, portraying the fits to corresponding data points, it becomes apparent that the curves associated with $T_p =323\,\hbox{K}$ and $333\,\hbox{K}$ exhibit overlapping behavior but differ in slope when contrasted with the overlapping curves for $T_p = 293\,\hbox{K}$, 303 K, and 313 K. Notably, experiments for the latter subset were conducted on a different day than those for the former. The observed deviation is hypothesized to variations in the dry ice pellets manufactured on two distinct days, resulting in differing permittivity of dry ice pellets. Consequently, this discrepancy manifests in distinct $B'$ values, representative of the slope of the curve for a given set of $A'$ and $\tau$.

To demonstrate a concrete correlation between the observed capacitance variation depicted in Fig. 6a and the thickness of the vapor layer existing between the dry ice pellet and the underlying substrate, the corresponding values, obtained at comparable time intervals, are juxtaposed in Fig. 6c. The relationship between capacitance variation and vapor layer thickness, as described by the fitted curve ($\Delta C = Ae^{-B\delta }$), bears resemblance to the numerical and experimental findings previously elucidated for a Teflon cylinder in Fig. 5b. Notably, while the constant *B* values in the fitting curves of Figs. 6c and 5b coincide, there exists a disparity in the constant *A* values between the two cases. This discrepancy is likely attributable to variations in permittivity values between the manufactured dry ice pellets and the Teflon cylinder discussed earlier, stemming from their distinct production processes and material characteristics. Nevertheless, the reasonable agreement between measured capacitance values for the dry ice pellet and the anticipated trend of variation with vapor layer thickness underscores the feasibility of correlating relative capacitance changes of the sensor with the separation distance between the Leidenfrost object and the underlying sensor. Expanding on the earlier discussion, it is demonstrated in this section that the variation of the sensor’s capacitance serves as an indicative measure of the vapor layer thickness beneath a disc-shaped dry ice pellet. The subsequent section systematically explores the quantified values of vapor layer thickness derived from capacitance data, considering various substrate temperatures.

#### Vapor layer thickness as a function of substrate temperature

In this section the variation of the initial value of the dimensionless vapor layer thickness ($\delta _0^*$) as a function of the dimensionless sublimation number ($\varepsilon ^*$) predicted in this work has been compared to that of the measured variation predicted by the OCT technique^23^. The vapor layer thickness from Eqs. (5) and (6) is made non-dimensional using the pellet diameter which result in the following expression:

9 $$\begin{aligned} \delta ^{*}(t)={\varepsilon ^{*}}^{\frac{1}{4}}\left( 1-\frac{t}{\tau }\right) ^{-\frac{1}{3}}, \end{aligned}$$

where

10 $$\begin{aligned} \varepsilon ^{*}= \frac{3}{8}\frac{\bar{\upmu _v}\bar{k_v}\Delta T}{L\rho _sgH_i\rho _{v,sat}D_i^2}. \end{aligned}$$

At the initial time, i.e. when $t\rightarrow 0$, the initial value of the vapor layer thickness ($\delta _0^*$) is function only of the dimensionless parameter, $\varepsilon ^*$ , as shown below equation:

11 $$\begin{aligned} \delta _0^* ={\varepsilon ^{*}}^{\frac{1}{4}} . \end{aligned}$$

**Figure 7:**
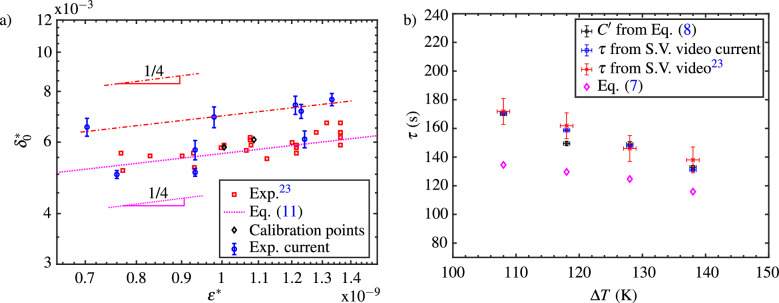
(**a**) Initial value of the dimensionless vapor layer thickness as a function of the sublimation number. (**b**) Lifetime of a disc-shaped dry ice pellet placed on the sensor as a function of the temperature difference between the substrate and the pellet.

The variation of $\delta _0^*$ with $\varepsilon ^{*}$ is shown in Fig. 7a. The square data points correspond to the values of the initial vapor layer thickness measured using OCT technique for different substrate temperatures which in turn mean different values of $\varepsilon ^*$ (see Eq. (10)). The corresponding values predicted using Eq. (11) is shown by the dotted line in Fig. 7a. In order to evaluate the value of the initial value of the vapor layer thickness from the capacitance fit given by Eq. (8) at $t=0$ using the known $A'$ and $B'$ values for different substrate temperatures, it is imperative to calibrate for the value of the parameter *B* in the equation $B' = B\delta _0$ with the known value $\delta _0$. In this case, measured values using the OCT technique serves as calibration points as shown by the diamond-shaped symbol in Fig. 7a. The two calibration points correspond to the values of $\delta _0^*$ values evaluated for substrate temperatures of $T_p = 323$ K and $T_p = 313$ K each for the respective groups of overlapping curves in Fig. 6b. Using these calibrated values, the values of $\delta _0 = B'/B_c$ for the experiments with distinct substrate temperatures, ranging from $T_p = 293$ K to $T_p=333$ K in the steps of 10 K is determined where $B_c$ represents the calibrated value. The corresponding values of $\delta _0^*$ evaluated in this manner for different values of $\varepsilon ^*$ in the present work is depicted using circular symbols in Fig. 7a. The 95% confidence interval of the parameter $B'$ has been considered in evaluating the error in the values of $\delta _0^*$. It can be seen from Fig. 7a that while some of the data points of the dimensionless vapor layer thickness derived from the capacitance values are in reasonable agreement with that of the measured values using OCT technique and the values predicted using Eq. (11), others deviate by $\sim 18\%$ from the latter two. This deviation might originate from the variations of the packing density of the dry ice pellet used in calibration versus the ones used in experiments leading to deviations in permittivity of the dry ice pellet between different experiment runs. In spite of these deviations, it can be seen that these values seem to vary with $\varepsilon ^*$ in a way that is similar to the variation measured by the OCT technique and predicted by Eq. (11), i.e. the dimensionless vapor layer thickness is proportional to $\varepsilon ^{*1/4}$ for a disc shaped dry ice pellet. To further demonstrate the feasibility of the capacitive sensor employed in this study, the following section analyzes the pellet’s lifetime as a function of substrate temperatures. Importantly, this parameter remains independent of the permittivity of the dry ice pellet, in contrast to the analysis presented in this section.

#### Lifetime of pellet as a function of substrate temperature

In the relation of the temporal evolution of $\Delta C$ given by the Eq. (8), the fitting parameter $C'$ indicates the lifetime of the dry ice pellet for a given substrate temperature. When the dry ice pellet reaches the end of its lifetime, i.e. when $t\rightarrow C'$, the value of capacitance measured by the sensor approaches its base value and consequently, the value of $\Delta C$ approaches zero as shown in Fig. 6a. The lifetime of the pellet determined from the value of $C'$ for four different substrate temperatures is shown in Fig. 7b as a function of the temperature difference between the substrate and the pellet ($\Delta T = T_p - T_s$). Additionally, the lifetime evaluated from the video captured by a camera of the present work, of our previous work^23^, and the lifetime predicted using Eq. (7) for similar substrate temperatures, is also shown for comparison. The error bars in the values of $C'$ correspond to its 95% confidence interval, and the error bars for the lifetime evaluated from the videos are determined by the resolution of the cameras. Towards the end of its lifetime the pellet shrinks and sublimates rapidly, and the corresponding gray scale intensity values for the images captured are noisy due to the limited frame and resolution of video cameras used in^23^, and therefore, the error bars corresponding to the lifetime pellet evaluated from this work have large error bars as opposed to the present work. The error bars in the value of substrate temperatures for which the pellet’s lifetime is evaluated is based on the vapor cooling of the substrate caused by the dry ice pellet, measured in its vicinity, over the period of its lifetime. As seen from Fig. 7b, while the values of the pellet’s lifetime evaluated the parameter $C'$ and the ones evaluated from the video cameras reasonably agree well with each other, Eq. (7) underestimates the pellet’s lifetime for all substrate temperatures. This underestimation of the predicted lifetime can result from two factors. First, is related to the assumption of the isothermal temperature of the substrate on which the dry ice is sublimating. This assumption however is questionable for substrates having low thermal conductivity as described in the work of Limbeek et al. ^34^. Failing to account for the effect of vapor cooling results in an overestimation of the substrate temperature and heat transfer rate, and in turn underestimation of the lifetime of the pellet. Second major factor leading to the underestimation of the pellet’s lifetime predicted by the model is the assumption of constant pellet diameter during the sublimation process. While this assumption holds for majority of pellet’s lifetime, it breaks down towards the end of sublimation where the pellet shrinks rapidly in diameter^23^. Since the heat transfer rate from the substrate to the pellet is proportional to the bottom surface area of the pellet, the assumption constant pellet diameter results in overestimation of the heat transfer rate leading to the underestimation of pellet’s lifetime.

## Conclusion

This work proposes a non-invasive capacitive method for investigating the Leidenfrost phenomenon, employing a sensor with a double comb structure of copper electrodes. The results of the analytical modeling presented in this study establish a design guideline for comb finger spacing. The study characterizes capacitive sensors for their output capacitance at controlled distances from a Teflon cylinder, showing an exponential decrease in capacitance with distance. The study also investigates the influence of sensor substrate material and finger spacing on the sensor’s capacitance output. The substrate material has minimal impact, while decreasing finger spacing enhances the rate of capacitance decrease. The sensor’s capability is demonstrated by measuring its output during the sublimation of a dry ice pellet. Subsequently, the vapor layer thickness values derived from the sensor’s capacitance output are compared with benchmark data obtained from Optical Coherence Tomography (OCT). The observed capacitance decrease aligns with the temporal increase in vapor layer thickness, consistent with OCT results. Initial values of dimensionless vapor layer thickness from capacitance measurements at different substrate temperatures are compared with OCT values, showing a considerable deviation but similar overall variation. Whereas the predicted dry ice pellet lifetime from capacitance values agrees reasonably well with observations from camera acquisitions during sublimation. While the sensor is sensitive to impurities and condensation, these practical limitations are associated with the Leidenfrost object and the experimental setup, not the fundamental sensor principle. Thus, the capacitive sensors introduced in this work hold potential for further exploration, refinement, and application in quantifying Leidenfrost vapor layers.

## Supplementary Information

Supplementary Figure 1.

## Data Availability

Data will be made available by the corresponding authors upon prior request.
