# Supplementary material for: A non-invasive capacitive sensor to investigate the Leidenfrost phenomenon: a proof of concept study
Source: Sci Rep. 2024 May 8;14:10565. doi: 10.1038/s41598-024-61222-z (PMC11634904; doi:10.1038/s41598-024-61222-z)
Supplement: Supplementary file 1 — Supplementary Figure 1. [file 41598_2024_61222_MOESM1_ESM.pdf]

Supplementary information for: A non-invasive capacitive sensor to investigate the Leidenfrost phenomenon: A proof of concept study

The images of the experimental setup utilized in the characterization study and for exploring the Leidenfrost phenomenon with the capacitive sensor are depicted in supplementary Fig. 1a) and supplementary Fig. 1b), respectively. These images represent the actual configuration of the experimental setup, which is schematically depicted in Fig. 3a) and Fig. 3b) of the manuscript.

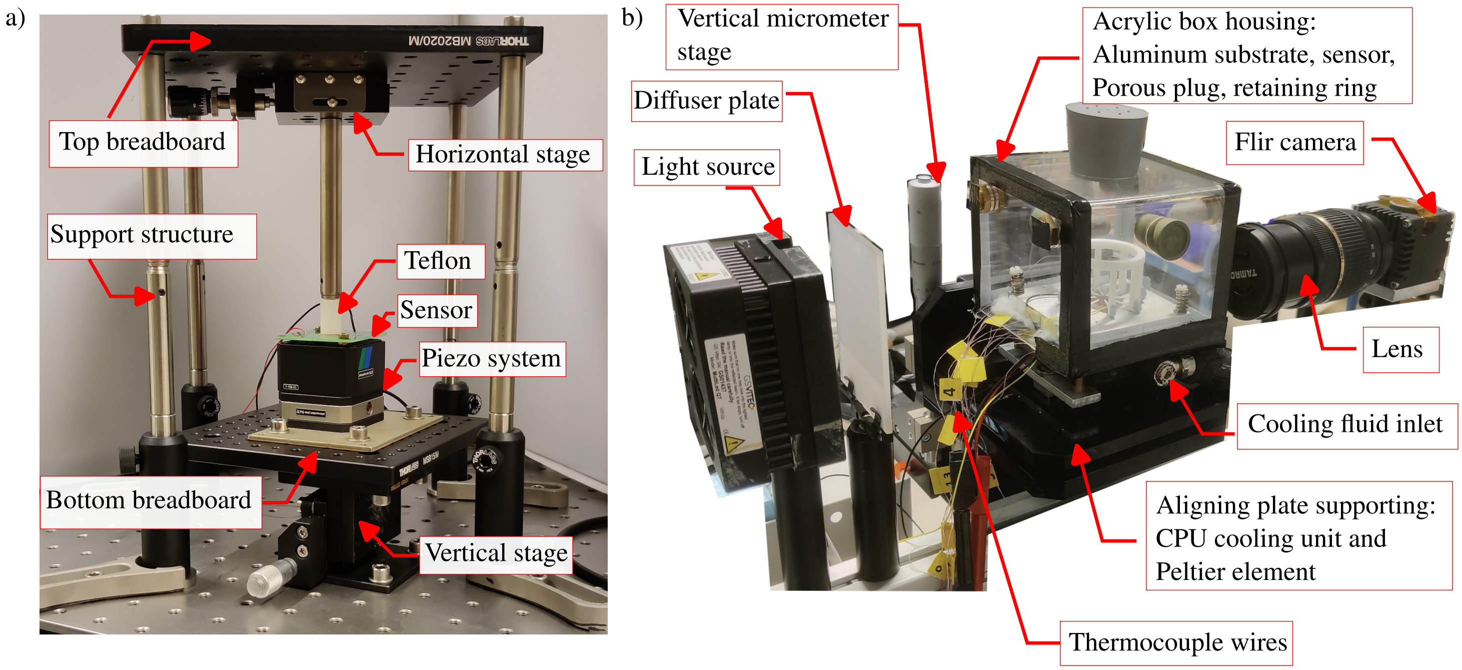

Supplementary figure 1. a) The experimental setup to characterize sensor capacitance as a function of the distance between the sensor and the Teflon cylinder. b) The experimental setup to test the feasibility of the capacitive sensor for investigating the Leidenfrost phenomenon of a dry ice pellet.
